# Radiochemotherapy with or without cetuximab for unresectable esophageal cancer: final results of a randomized phase 2 trial (LEOPARD-2)

**DOI:** 10.1007/s00066-020-01646-4

**Published:** 2020-06-12

**Authors:** Dirk Rades, Tobias Bartscht, Peter Hunold, Heinz Schmidberger, Laila König, Jürgen Debus, Claus Belka, Nils Homann, Patrick Spillner, Cordula Petersen, Thomas Kuhnt, Rainer Fietkau, Karsten Ridwelski, Kerstin Karcher-Kilian, Anne Kranich, Sofia Männikkö, Steven E. Schild, Annett Maderer, Markus Moehler

**Affiliations:** 1 Department of Radiation Oncology, University of Lübeck, Ratzeburger Allee 160, 23562 Lübeck, Germany; 2 Department of Hematology and Oncology, University of Lübeck, Lübeck, Germany; 3 Department of Radiology and Nuclear Medicine, University of Lübeck, Lübeck, Germany; 4 Department of Radiation Oncology, Johannes-Gutenberg University Mainz, Mainz, Germany; 5 Department of Radiation Oncology, University of Heidelberg, Heidelberg, Germany; 6 Department of Radiation Oncology, Ludwig-Maximillians University, Munich, Germany; 7 Medical Department II, Klinikum Wolfsburg, Wolfsburg, Germany; 8 Department of Radiation Oncology, Eberhard-Karls University, Tübingen, Germany; 9 Department of Radiotherapy and Radiation Oncology, University Medical Center Hamburg-Eppendorf, Hamburg, Germany; 10 Department of Radiation Oncology, University Hospital Leipzig, Leipzig, Germany; 11 Department of Radiation Oncology, Friedrich-Alexander University Erlangen-Nürnberg, Erlangen, Germany; 12 Department of General and Visceral Surgery, Klinikum Magdeburg, Magdeburg, Germany; 13 Practice for Gastroenterology, Diabetology, Oncology and Hematology Lübeck, Lübeck, Germany; 14 Gesellschaft für Studienmanagement und Onkologie mbH, Hamburg, Germany; 15 Pharma Ltd, Turku, Finland; 16 Department of Radiation Oncology, Mayo Clinic Scottsdale, AZ, USA; 17 1st Department of Internal Medicine, Johannes Gutenberg-University Mainz, Mainz, Germany

**Keywords:** Esophageal cancer, Definitive treatment, EGFR antibody, Efficacy, Feasibility

## Abstract

**Purpose:**

To investigate the efficacy and toxicity of cetuximab when added to radiochemotherapy for unresectable esophageal cancer.

**Methods:**

This randomized phase 2 trial (clinicaltrials.gov, identifier NCT01787006) compared radiochemotherapy plus cetuximab (arm A) to radiochemotherapy (arm B) for unresectable esophageal cancer. Primary objective was 2‑year overall survival (OS). Arm A was considered insufficiently active if 2‑year OS was ≤40% (null hypothesis = H_0_), and promising if the lower limit of the 95% confidence interval was >45%. If that lower limit was >40%, H_0_ was rejected. Secondary objectives included progression-free survival (PFS), locoregional control (LC), metastases-free survival (MFS), response, and toxicity. The study was terminated early after 74 patients; 68 patients were evaluable.

**Results:**

Two-year OS was 71% in arm A (95% CI: 55–87%) vs. 53% in arm B (95% CI: 36–71%); H_0_ was rejected. Median OS was 49.1 vs. 24.1 months (*p* = 0.147). Hazard ratio (HR) for death was 0.60 (95% CI: 0.30–1.21). At 2 years, PFS was 56% vs. 44%, LC 84% vs. 72%, and MFS 74% vs. 54%. HRs were 0.51 (0.25–1.04) for progression, 0.43 (0.13–1.40) for locoregional failure, and 0.43 (0.17–1.05) for distant metastasis. Overall response was 81% vs. 69% (*p* = 0.262). Twenty-six and 27 patients, respectively, experienced at least one toxicity grade ≥3 (*p* = 0.573). A significant difference was found for grade ≥3 allergic reactions (12.5% vs. 0%, *p* = 0.044).

**Conclusion:**

Given the limitations of this trial, radiochemotherapy plus cetuximab was feasible. There was a trend towards improved PFS and MFS. Larger studies are required to better define the role of cetuximab for unresectable esophageal cancer.

## Introduction

The prognosis of patients with locally advanced esophageal cancer is poor and requires improvement that may be achieved with the addition of new drugs [1]. For definitive and neoadjuvant treatment of locally advanced disease, radiochemotherapy with cisplatin and 5‑fluorouracil (5-FU) has been the standard regimen for more than 20 years [2]. The combination of radiotherapy and carboplatin/paclitaxel has been popular in the neoadjuvant setting since publication of a randomized trial in 2012 [3]. Combinations of radiotherapy or radiochemotherapy with newer systemic therapies such as antibodies to the epidermal growth factor receptor (EGFR) provide additional options. Overexpression of EGFR is frequent and associated with a poor prognosis in patients with squamous cell carcinoma (SCC) of the esophagus or adenocarcinoma of the gastroesophageal junction [4–6]. For cetuximab, a radiosensitizing effect was shown in preclinical studies and anticancer activity in small clinical studies [7–11]. These data led to the present trial that investigated the efficacy and feasibility of cetuximab added to radiochemotherapy for unresectable esophageal cancer.

## Patients and methods

This multicenter open-label, randomized phase 2 trial evaluated radiochemotherapy plus cetuximab in patients treated for unresectable esophageal cancer between 09/2011 and 12/2016. It was approved by the local ethic committees (leading committee: University of Lübeck, reference: 11-104) and performed in accordance with the Helsinki Declaration. It was registered at clinicaltrials.gov (identifier: NCT01787006). Eligible patients had histologically confirmed unresectable esophageal cancer. Resectability was determined by a surgeon prior to randomization. Inclusion and exclusion criteria are listed in Table 1.

**Table 1:** Inclusion and exclusion criteria

**Inclusion criteria** *Patients who met**** all of the following criteria**** could be enrolled into the study:* – Signed written informed consent – Male or female aged between 18 and 75 years; patients >75 years if Karnofsky performance score is ≥80 – Histologically proven squamous cell carcinoma or adenocarcinoma of the esophagus, which was not curatively resectable^a^ – Karnofsky performance score ≥70 – Women of child-bearing potential must have a negative pregnancy test. – Adequate cardiac, pulmonary, and ear function – Adequate bone marrow function – Adequate liver function – Adequate renal function – No known allergy against chimeric antibodies. – Effective contraception for both male and female patients if the risk of conception existed
**Exclusion criteria** *Patients who met ****any of the following criteria**** were not allowed to be enrolled into the study:* – Distant metastasis (M1b) – Previous treatment of esophageal cancer – Previous exposure to monoclonal antibodies and/or epidermal growth factor receptor(EGFR)-targeted therapy – Other previous malignancy with exception of a history of a previous curatively treated basal cell carcinoma of the skin or pre-invasive carcinoma of the cervix – Serious concomitant disease or medical condition – Forced expiratory volume in the first second (FEV_1_) <1.1 l – Clinically relevant coronary artery disease or a history of myocardial infarction within the last 12 months or left ventricular ejection fraction below the institutional range of normal – Any active dermatological condition grade >1 – Contraindications to receiving cisplatin, 5‑fluorouracil, or cetuximab – Concurrent treatment with other experimental drugs or participation in another clinical trial with any investigational drug within 30 days prior to study screening – Pregnancy or lactation – Known active drug abuse/alcohol abuse – Social situations limiting the compliance with the study requirements

^a^Resectability was defined by a surgeon prior to randomization. The tumor was considered unresectable due to T‑stage, N‑stage, performance/nutritional status, comorbidity (pulmonary function, other), tumor location upper third of the esophagus, relation to other organs/structures, or other reasons

Patients were randomly assigned to radiochemotherapy plus cetuximab (experimental group, arm A) or radiochemotherapy alone (control group, arm B). Stratification was based on histology (SCC vs. adenocarcinoma), Karnofsky performance score (80–100% vs. 70%), and tumor stage (T1-3N0‑1 vs. T4 and/or N2 and/or M1a) [12]. Both histologies were allowed, since at the time of trial initiation, the standard treatment was the same.

### Treatments

**Fig. 1:**
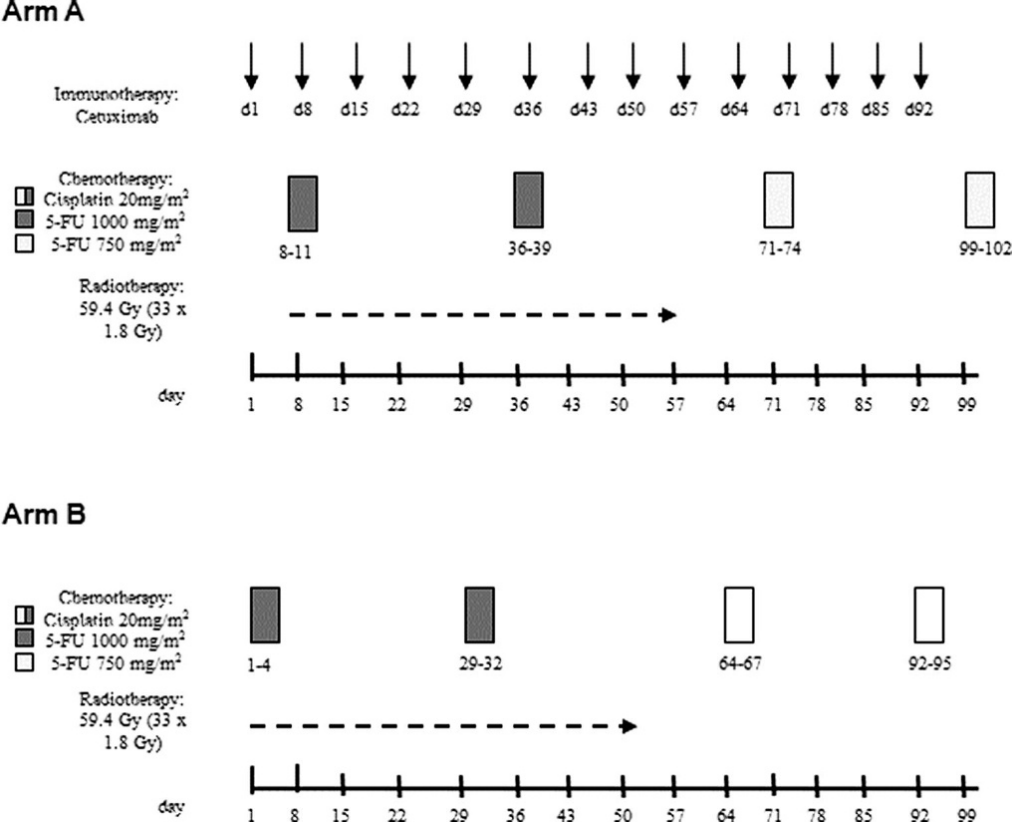
Flowchart of the treatments administered in this trial. *5‑FU* 5-fluorouracil

Radiotherapy was performed with photons from a linear accelerator using 3D treatment planning. Initially, 50.4 Gy in 28 fractions was planned for primary tumor plus locoregional lymph nodes. Reevaluation assessing resectability was performed after 4–4.5 weeks. If resectability was achieved and the patient agreed to surgery, radiotherapy was stopped after 45 Gy and the patient underwent surgery. This applied to 8 patients (25%) of the experimental group and 17 patients (47%) of the control group (*p* = 0.079, Fisher’s exact test). If resectability was not achieved or the patient refused surgery, radiotherapy was continued until 50.4 Gy and followed by a boost of 9.0 Gy to primary tumor and involved lymph nodes (Fig. 1). Treatment planning and quality assurance were performed according to the standard operating procedures of the contributing centers. Generally, the initial clinical target volume (CTV) included the gross tumor volume (GTV) plus margins of 3–5 cm in the superior–inferior direction and 1 cm in the lateral and anterior–posterior directions. The CTV for the boost included the GTV plus margins of 2 cm in the superior–inferior direction and 1 cm in lateral and anterior–posterior directions. For the involved lymph nodes, a GTV-to-CTV margin of 0.5–1.0 cm was suggested. The margin from the CTV to the planning target volume (PTV) was 0.5–1.0 cm. In accordance with the QUANTEC (Quantitative Analyses of Normal Tissue Effects in the Clinic) data, the mean doses for heart, lung, liver, and kidney (bilateral) should be <26 Gy, ≤7 Gy, <30–32 Gy, and <15 Gy, respectively [13]. Moreover, the dose to the spinal cord should not exceed 45 Gy. A brachytherapy boost was not implemented in the protocol, since this is not a standard therapy for the primary treatment of esophageal cancer. However, it can be a reasonable option for a local recurrence or symptom relief in a palliative situation [14].

**Fig. 2:**
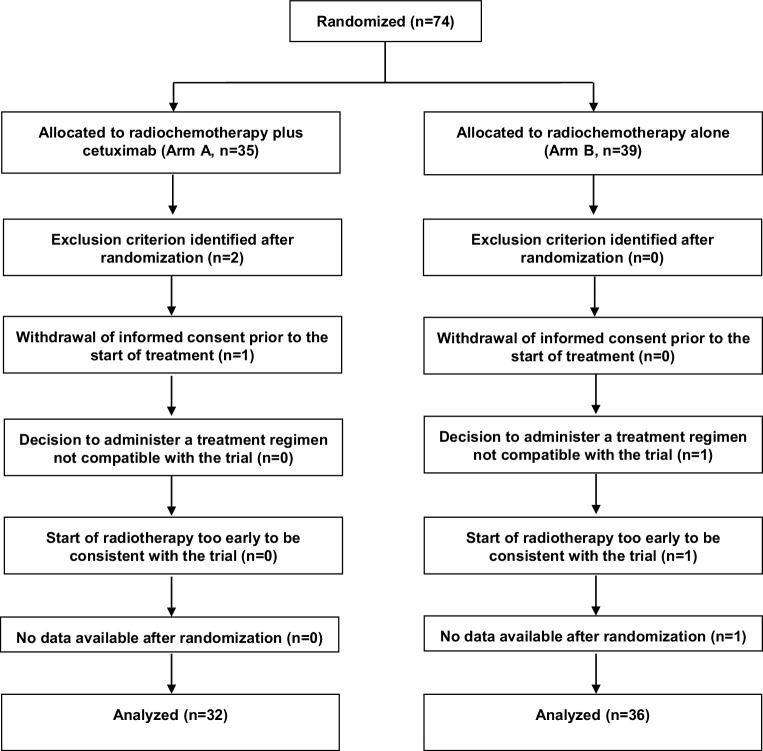
Consolidated Standards of Reporting Trials (CONSORT) diagram

Two courses of 5‑FU (1000 mg/m^2^/d) were administered as a continuous infusion over 96 h during the first and fifth weeks of radiotherapy (Fig. 1; [2, 15]). Two cycles of 750 mg/m^2^/d of 5‑FU (over 96 h) were administered after radiotherapy, 5 and 9 weeks after the second course. Cisplatin (20 mg/m^2^/d) was administered after saline hydration as an intravenous bolus over 60 min on the same days as 5‑FU. Patients received antiemetic therapy prior to cisplatin, including 5HT3 antagonists and dexamethasone.

Cetuximab was administered as an intravenous infusion with a loading dose of 400 mg/m^2^ over 120 min (day 1), followed by weekly doses of 250 mg/m^2^ over 60 min for a total of 14 weeks (Fig. 1). Patients were pretreated with antihistamines and glucocorticoids.

### Statistical considerations

The primary objective was to determine the 2‑year OS rate of the two cohorts. OS was referenced from the day of randomization and analyzed using the Kaplan–Meier method and the univariate Cox proportional hazards method. For the primary hypothesis of 2‑year OS, OS rate and 95% confidence interval (CI) were calculated for the 2‑year timepoint. The 2‑year OS of 40% for the reference group was expected based on prior studies [2, 16, 17]. If the observed 2‑year OS rate was ≤40% (null hypothesis H_0_) for the experimental therapy (arm A), this therapy would be deemed insufficiently active to pursue in further research. The alternative hypothesis (H_1_) was that adding cetuximab would result in 2‑year OS >40%. The decision for rejection of H_0_ was based on the 95% CI of the 2‑year OS. H_0_ could be rejected if the lower limit of the 95% CI was >40%. If the lower limit was >45%, radiochemotherapy plus cetuximab would be considered a promising enough therapy to justify further investigation. The probability of accepting the experimental therapy as promising (2-year OS >45%) when the true OS rate was ≤40%, was 5% (type I error). The probability of rejecting the experimental therapy as insufficiently active (≤40%) when the true OS rate was promising (>45%), was 20% (type II error, power of 80%).

Secondary objectives included determination of 1‑year OS, progression-free survival (PFS), locoregional control (LC), metastases-free survival (MFS), overall response (OR; RECIST v11 [18]), and toxicities (CTCAE v4.03 [19]). In addition, OS, PFS, and MFS were assessed irrespectively of specific timepoints. For PFS, the event was defined as first occurrence of radiologically proven or clinical progression or death due to progressive disease. Locoregional failure was defined as progressive primary tumor and/or regional lymph nodes on endoscopy, endoscopic ultrasound, or computed tomography. For MFS, the event was defined as first occurrence of distant metastasis. Both groups were compared for these endpoints using the Kaplan–Meier method and the log-rank test. Differences between OR rates were compared with the chi-square test. If frequencies in one group were ≤5, Fisher’s exact test was used. The comparisons of toxicities were performed with the Fisher’s exact test.

When using a standard single-stage phase 2 design according to Fleming [20], 124 evaluable patients were required to determine efficacy. The standard treatment (control group) served to reduce some of the result variability typically encountered in single-arm phase 2 trials. To cover potential dropouts, 134 patients should be recruited.

The trial was terminated after randomization of 74 patients (35 arm A, 39 arm B) due to slow accrual. Sixty-eight patients (32 arm A, 36 arm B) were evaluable for efficacy and toxicity (Fig. 2 and Table 2). This number of patients was sufficient to detect a difference of approximately 33% with a statistical power of 80% at a two-sided significance level of 0.05. Median follow-up was 18 (0–61) months in the entire cohort, and 26 (7–61) months in patients alive at last contact.

**Table 2:** Patient and tumor characteristics of the experimental group (*n* = 32) and the control group (*n* = 36)

	Experimental group (arm A) *N* (%)	Control group (arm B) *N* (%)
*Age (years)*		
Median (range)	65 (44–80)	64 (49–79)
*Gender*		
Male	22 (68.8)	30 (83.3)
Female	10 (31.3)	6 (16.7)
*Ethnic origin*		
Caucasian	32 (100)	36 (100)
*Histology*		
Squamous cell carcinoma	27 (84.4)	28 (77.8)
Adenocarcinoma	5 (15.6)	8 (22.2)
*Histologic grading*		
G1	0 (0.0)	1 (2.8)
G2	16 (50.0)	18 (50.0)
G3	11 (34.4)	10 (27.8)
Gx	5 (15.6)	7 (19.4)
*Main tumor location*		
Lower third	9 (28.1)	15 (41.7)
Middle third	14 (43.8)	11 (30.6)
Upper third	9 (28.1)	8 (22.2)
Unknown	0 (0.0)	2 (5.6)
*T‑category *[15]		
T1	1 (3.1)	1 (2.8)
T2	2 (6.3)	0 (0.0)
T3	20 (62.5)	20 (55.6)
T4	8 (25.0)	13 (36.1)
Tx	1 (3.1)	2 (5.6)
*N‑category *[15]		
N0	7 (21.9)	8 (22.2)
N1	14 (43.8)	14 (38.9)
N2	7 (21.9)	7 (19.4)
N3	3 (9.4)	3 (8.3)
N x	0 (0.0)	3 (8.4)
N +	1 (3.1)	1 (2.8)
*M‑category *[15]		
M0	31 (96.9)	35 (97.2)
M1a	0 (0.0)	1 (2.8)
Mx	1 (3.1)	0 (0.0)

## Results

Two-year OS rates were 71% in arm A (95% CI: 55–87%) vs. 53% in arm B (95% CI: 36–71%). Since the lower limit of the 95% CI of the 2‑year OS rate in arm A was 55% (>40%), H_0_ was rejected. Since this value was also >45%, radiochemotherapy plus cetuximab was deemed promising.

**Fig. 3:**
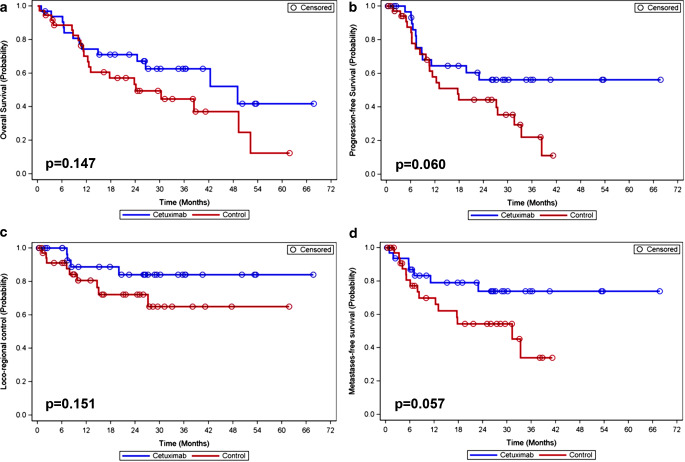
Comparison of the two treatment groups (Kaplan–Meier analysis and log-rank test) with respect to overall survival (**a**), progression-free survival (**b**), locoregional control (**c**), and metastases-free survival (**d**)

Median OS was 49.1 months in arm A and 24.1 months in arm B (*p* = 0.147); 1‑year OS rates were 74% and 70%, respectively (Fig. 3). Hazard ratio (HR) for death was 0.60 (95% CI: 0.30–1.21). Since less than half of the patients in arm A experienced progression, the median time of PFS could not be estimated; in arm B, median time of PFS was 17.6 months (*p* = 0.060). PFS was 64% vs. 58% at 1 year and 56% vs. 44% at 2 years (Fig. 3). HR for progression was 0.51 (95% CI: 0.25–1.04). LC was 89% vs. 81% at 1 year and 84% vs. 72% at 2 years (*p* = 0.151; Fig. 3). HR for locoregional failure was 0.43 (95% CI: 0.13–1.40). Since less than 50% of patients in arm A experienced distant metastasis, the median time of MFS could not be estimated; in arm B, median time of MFS was 31.3 months (*p* = 0.057). MFS was 79% vs. 70% at 1 year and 74% vs. 54% at 2 years (Fig. 3). HR for distant metastasis was 0.43 (95%-CI: 0.17–1.05). OR rates were 81.3% vs. 69.4% (*p* = 0.262).

All 68 patients who received at least one dose of study medication experienced at least one adverse event (AE). Of all AEs when counted separately, 74.5% were mild or moderate. Significantly more AEs (any grade) were observed in arm A for leukopenia (50.0% vs. 22.2%, *p* = 0.023), hypocalcemia (28.1% vs. 5.6%, *p* = 0.019), hypomagnesemia (40.6% vs. 8.3%, *p* = 0.003), acneiform rash (34.4% vs. 0%, *p* < 0.001), radiation dermatitis (28.1% vs. 2.8%, *p* = 0.005), maculopapular rash (21.9% vs. 2.8%, *p* = 0.022), and allergic reactions (12.5% vs. 0%, *p* = 0.044). Non-significantly higher rates were found in arm A for thrombopenia (34.4% vs. 19.4%, *p* = 0.182), hypopotassemia (50.0% vs. 33.3%, *p* = 0.219), oral mucositis (25.0% vs. 11.1%, *p* = 0.203), weight loss (28.1% vs. 8.3%, *p* = 0.054), and pneumonitis (6.3% vs. 2.8%, *p* = 0.598). Non-significantly higher rates (any grade) were observed in arm B for fatigue (50.0% vs. 28.1%, *p* = 0.085), constipation (30.6% vs. 18.8%, *p* = 0.401), and lung infection (25.0% vs. 9.4%, *p* = 0.118).

Twenty-six patients (81.3%) in arm A and 27 patients (75.0%) in arm B (*p* = 0.573) experienced at least one AE grade ≥3 (Table 3). A significant difference was found only for allergic reactions (12.5% vs. 0%, *p* = 0.044). All allergic reactions were managed and resolved without sequelae. Two patients in arm B died during the treatment phase due to underlying disease and cardiovascular disorders, respectively.

**Table 3:** Severe adverse events (grade ≥3) occurring in more than 1 patient (safety analysis set, *n* = 68)

	Experimental group (arm A) *N* (%)	Control group (arm B) *N* (%)	*p*-value^a^
Lung infection	3 (9.4)	8 (22.2)	0.196
Leukopenia	7 (21.9)	4 (11.1)	0.326
Anemia	4 (12.5)	7 (19.4)	0.521
Esophagitis	6 (18.8)	5 (13.9)	0.744
Dysphagia	4 (12.5)	3 (8.3)	0.699
Thrombopenia	4 (12.5)	2 (5.6)	0.410
Hypopotassemia	3 (9.4)	2 (5.6)	0.660
Neutropenia	2 (6.3)	3 (8.3)	1.000
Allergic reaction	4 (12.5)	0 (0.0)	0.044
Diarrhea	3 (9.4)	1 (2.8)	0.336
Nausea	1 (3.1)	3 (8.3)	0.616
Thromboembolic event	2 (6.3)	2 (5.6)	1.000
Radiation dermatitis	3 (9.4)	0 (0.0)	0.099
Dyspnea	2 (6.3)	1 (2.8)	0.598
GGT increased	2 (6.3)	1 (2.8)	0.598
Hypomagnesemia	3 (9.4)	0 (0.0)	0.099
Acneiform rash	3 (9.4)	0 (0.0)	0.099
Sepsis	1 (3.1)	2 (5.6)	1.000
Dehydration	0 (0.0)	2 (5.6)	0.494
Device-related infection	2 (6.3)	0 (0.0)	0.218
Fatigue	0 (0.0)	2 (5.6)	0.494
Gastric ulcer	1 (3.1)	1 (2.8)	NC
Hypertension	2 (6.3)	0 (0.0)	0.218
Infection	1 (3.1)	1 (2.8)	NC
Pleural effusion	1 (3.1)	1 (2.8)	NC
Syncope	1 (3.1)	1 (2.8)	NC
Vomiting	0 (0.0)	2 (5.6)	0.494
Weight loss	1 (3.1)	1 (2.8)	NC

*GGT* gamma-glutamyltransferase, *NC* not calculated

^a^*p*-values were calculated with the Fisher’s exact test

In arm A, cetuximab was discontinued after the loading dose due to allergic reactions in 4 patients. In the remaining 28 patients, cetuximab was stopped after 6 doses, since patients went to surgery. In another 6 patients, cetuximab was discontinued (reason not specified) after 7 (*n* = 4), 8 (*n* = 1), and 11 (*n* = 1) doses, respectively.

The proportions of patients requiring a reduction of cisplatin during cycles 1–4 were 3.4%, 10.7%, 18.9%, and 8.3%, respectively, in arm A vs. 5.6%, 14.3%, 27.3%, and 10.0%, respectively, in arm B. Toxicity-associated dose reductions of 5‑FU in cycles 1–4 were required by 3.4%, 11.5%, 13.3%, and 8.3% of patients in arm A vs. 2.8%, 14.8%, 10.0%, and 0% of patients in arm B, respectively. Using the Fisher’s exact test, the differences between the two groups regarding the reduction of cisplatin and 5‑FU were not significant. In arm A, 3 patients (9.4%) who experienced allergic reactions to cetuximab did not receive radiotherapy. In the irradiated patients, interruption of radiotherapy for >7 days was required in 0% (arm A) and 11.1% (arm B) of patients (*p* = 0.122, Fisher’s exact test), and a reduction of the planned radiotherapy dose in 10.3% and 19.4%, respectively (*p* = 0.492).

## Discussion

During recent years, a considerable amount of research has been performed to improve the prognoses of patients with esophageal cancer [21–24]. The present trial investigated the impact of adding cetuximab to radiochemotherapy for unresectable esophageal cancer. In the experimental arm (arm A), 2‑year OS was 71% (95% CI: 55–87%) compared to 53% in arm B (95% CI: 36–71%). The lower limit of the 95% CI met the trial criteria. Therefore, H_0_ was rejected and radiochemotherapy plus cetuximab was deemed promising. Two-year OS was also higher in arm B than in the reference studies [2, 16, 17]. However, the 95% CI in arm B included the threshold of 40%. Median OS was longer in arm A (49.1 vs. 24.1 months), although significance was not achieved.

Our trial suggested a trend towards improved PFS and MFS when cetuximab was added. Conflicting results were reported from other studies. In the SCOPE‑1 trial, patients were randomized to radiochemotherapy with 50 Gy in 25 fractions plus four cycles of cisplatin 60 mg/m^2^/d1 and capecitabine 625 mg/m^2^ twice daily/d1-21, or to the same regimen plus cetuximab (400 mg/m^2^/d1 followed by 250 mg/m^2^ weekly) [25]. In 2017, long-term results of this trial were published [26]. Median OS was 34.5 months without and 24.7 months with cetuximab (*p* = 0.137). Median PFS was 24.1 vs. 15.9 months (*p* = 0.114).

The RTOG 0436 trial compared definitive radiochemotherapy with 50.4 Gy in 28 fractions plus weekly cisplatin (50 mg/m^2^) and paclitaxel (25 mg/m^2^) with or without cetuximab for unresectable esophageal cancer [27]. OS rates at 2 and 3 years were 45% and 34% with vs. 44% and 28% without cetuximab (*p* = 0.47). Local failure rates at 2 and 3 years were 47% and 49% vs. 49% and 49% (*p* = 0.65).

A third randomized trial adding cetuximab to radiochemotherapy in patients with resectable esophageal cancer was favorable [28]. It compared chemotherapy (two cycles of 75 mg/m^2^ docetaxel and 75 mg/m^2^ cisplatin) followed by radiochemotherapy (45 Gy in 25 fractions plus weekly 20 mg/m^2^ docetaxel and 25 mg/m^2^ cisplatin) and surgery to the same regimen plus cetuximab (neoadjuvant: 250 mg/m^2^ weekly; adjuvant: 500 mg/m^2^ every second week). Median PFS was 2.9 years with and 2.0 years without cetuximab (*p* = 0.13). Median OS was 5.1 vs. 3.0 years (*p* = 0.055). Time to locoregional failure after R0 resection was significantly longer in the cetuximab group (*p* = 0.017).

The results of available prospective studies regarding toxicity were also conflicting. In the present trial, significantly more AEs (any grade) were found in the experimental arm for leucopenia, hypocalcemia, hypomagnesemia, acneiform rash, radiation dermatitis, maculopapular rash, and allergic reactions. When counted separately, the majority of all AEs were mild or moderate and managed without problems. A significant difference was observed for allergic reactions that resolved without sequelae. Rates of patients experiencing at least one AE grade ≥3 were not significantly different. This is consistent with the majority of prospective studies that rated radiochemotherapy plus cetuximab for esophageal cancer as feasible or well tolerated [15, 29–34].

In contrast, one randomized and four non-randomized studies concluded that radiochemotherapy plus cetuximab was associated with considerable toxicity [28, 30, 31, 35, 36]. The non-randomized studies used radiochemotherapy regimens different from the two most common regimens [2, 3]. In two studies, radiochemotherapy included cisplatin and irinotecan, in one study preoperative oxaliplatin/5-FU followed by postoperative docetaxel, and in one study induction chemotherapy with epirubicin, cisplatin, and capecitabine [37–40]. Therefore, the results of these studies are difficult to interpret. The SCOPE‑1 trial included two cycles of induction chemotherapy with cisplatin and capecitabine followed by radiochemotherapy with two concurrent cycles, which is also different from the most common regimens in use today [2, 3]. Moreover, the addition of cetuximab to radiochemotherapy resulted in increased toxicity in trials performed in patients with other cancer types such as head and neck cancer and anal carcinoma [41, 42].

Median OS of the cetuximab group in the LEOPARD-2 trial was better than in the SCOPE‑1 trial (49.1 vs. 24.7 months) [26]. This may be explained by the fact that in the LEOPARD-2 trial, no patient in the cetuximab arm required interruption of radiotherapy for >7 days, and only 9.4% of the patients did not receive radiotherapy. In the SCOPE‑1 trial, radiotherapy was not given to 19% of patients in the cetuximab arm, and interruptions of radiotherapy appeared more frequent in the cetuximab group. In head and neck cancer patients, interruptions of radiotherapy decrease disease control and survival [35, 43]. Moreover, the proportion of patients in the cetuximab group with SCC was higher in the LEOPARD-2 than in the SCOPE‑1 trial (84% vs. 71%). In a phase 1b/2 trial, SCC was associated with significantly better outcomes than adenocarcinoma regarding LC (96% vs. 74%, *p* < 0.001) and OS (58% vs. 25%, *p* = 0.001) [31]. In another prospective study, where all patients had SCC, CR was 69% and 2‑year PFS 75% [29]. Another important aspect is the low rate of acneiform skin reactions and rash (only 18%) in the SCOPE‑1 trial. This is important, since rash was associated with better outcomes in head and neck cancer patients receiving cetuximab [44]. In the LEOPARD-2 trial, 56% of patients experienced acneiform or maculopapular rash.

The results of the LEOPARD-2 trial regarding the addition of cetuximab also appeared more promising than in the RTOG 0436 trial (2-year OS: 71% vs. 45%) [26]. This may be explained by a considerably higher proportion of SCC in the cetuximab arm (84% vs. 37%) in the LEOPARD-2 trial. Interruptions of radiotherapy were not reported for the RTOG 0436 trial. Moreover, the chemotherapy regimen in the LEOPARD-2 trial was more intensive than in the RTOG 0436 trial. Although this regimen was associated with more grade ≥3 toxicities, many patients received the planned treatment due to intensive patient care including administration of chemotherapy and cetuximab under inpatient conditions.

Since different radiochemotherapy regimens were used in the available trials, the treatments were performed in different settings, and patient characteristics varied, it is difficult to compare the results of the LEOPARD-2 and other trials. When interpreting the results of the LEOPARD-2 trial, one should keep in mind its limitations, particularly the comparatively small sample size after premature termination. Additional limitations included lack of a central review, the fact that less than 20% of the patients had adenocarcinomas, no assessment of the expression of EGFR and mutations in the vast majority of the patients, and lack of standardized procedures of treatment planning and quality assurance. Moreover, one has to consider that the proportions of patients who underwent surgery after 45 Gy were different in both groups. This difference might have introduced a bias. Due to these limitations, the results of the present study appear less meaningful than the results of previous larger trials [25–28].

## Conclusion

Given the limitations of the LEOPARD-2 trial, radiochemotherapy with cisplatin/5-FU plus cetuximab is feasible for unresectable esophageal cancer. Although not statistically significant, there was a trend towards improved PFS and MFS. Larger studies are required to better define the role of the addition of cetuximab to radiochemotherapy for unresectable esophageal cancer.

## Abbreviations

CI: Confidence interval
CONSORT: Consolidated Standards of Reporting Trials
CTCAE: Common Terminology Criteria for Adverse Events
EGFR: Epidermal growth factor receptor
5‑FU: 5‑Fluorouracil
HR: Hazard ratio
LC: Locoregional control
MFS: Metastases-free survival
OR: Overall response
OS: Overall survival
PFS: Progression-free survival
RECIST: Response Evaluation Criteria in Solid Tumors
RTOG: Radiation Therapy Oncology Group
SCC: Squamous cell carcinoma
